# A Triggering Mechanism for Cyber-Attacks in Naval Sensors and Systems †

**DOI:** 10.3390/s21093195

**Published:** 2021-05-04

**Authors:** Walmor Cristino Leite Junior, Claudio Coreixas de Moraes, Carlos E. P. de Albuquerque, Raphael Carlos Santos Machado, Alan Oliveira de Sá

**Affiliations:** 1 Naval War College, Brazilian Navy, Rio de Janeiro 22290-255, Brazil; walmor.clj@outlook.com; 2 Naval Systems Analysis Centre, Brazilian Navy, Rio de Janeiro 20091-000, Brazil; coreixas@ufrj.br; 3 Ocean Technology Laboratory—LabOceano/COPPE, Federal University of Rio de Janeiro, Rio de Janeiro 21941-907, Brazil; 4 Institute of Computing, Fluminense Federal University, Niterói 24210-310, Brazil; carlosalbuquerque@id.uff.br (C.E.P.d.A.); raphaelmachado@ic.uff.br (R.C.S.M.); 5 National Institute of Metrology, Quality and Technology, Duque de Caxias 25250-020, Brazil; 6 Admiral Wandenkolk Instruction Center, Brazilian Navy, Rio de Janeiro 20180-003, Brazil

**Keywords:** cybersecurity, radar, automatic identification system, electronic chart display and information system, electronic attack, template matching

## Abstract

In the maritime sector, the integration of radar systems, Automatic Identification System (AIS) and Electronic Chart Display and Information System (ECDIS) through digital technologies enables several benefits to maritime operations, but also make ships prone to cyberattacks. In this context, this work investigates the feasibility of an attacker using a radar system or AIS as open door to remotely send commands to a cyber threat hosted on a ship, even if the ship’s systems are air gapped—i.e., are not connected to other networks. The received commands are intended to trigger a cyber threat located in the ship. Although the literature covers several analyzes on cyber risks and vulnerabilities in naval systems, it lacks exploiting mechanisms capable of acknowledging attack commands received through radar and AIS. To this end, this work proposes a triggering mechanism that uses a template matching technique to detect specific patterns transmitted by the attacker to the ship’s radar or AIS. The results show the effectiveness of the proposed technique as a tool to acknowledge the received attack commands and activate a malicious code previously installed on the ship. In the case of attacks on a radar system, the accuracy achieved by the proposed method is 0.90. In the case of attacks on an AIS/ECDIS setup it presents an accuracy of 0.93. In both cases the proposed mechanism maintains the due safety against accidental attack activations.

## 1. Introduction

Cyberattacks in the maritime environment have so far resulted in limited impacts and losses. However, due to the role of the maritime activities in the global integration and considering the growing digital transformation that pervades the sector, the cyber threats and associated losses are prone to grow. Scenarios of attacks to ports and ships can no longer be thought exclusively as consequences of physical offensives. It is necessary to consider the possibilities of having such attacks as consequences of cyber threats [1], with potential to cause impact in a world-class supply chain [2].

The development of ships is inevitably following the worldwide trends and boarding cutting-edge technologies of Industry 4.0, Internet of Things (IoT), smart sensors, and others [3]. The design of ships’ navigation systems is integrating Information Technology (IT), Operational Technology (OT) and digital naval sensors [3,4] to provide more efficiency and safety in the handling of vessels. However, at the same time that digital technologies enable several benefits to maritime operations, they also make ships prone to cyberattacks. The consequences of cyberattacks in the maritime environment can result in navigation accidents, pollution, serious economic costs, and losses of human lives.

To address this concern, the International Maritime Organization (IMO) published the Guidelines on Maritime Cyber Risk Management [5] which sparked a global movement towards strengthening cybersecurity in the maritime environment. The guidelines strongly recommend the adoption of early efforts by entities operating in the maritime sector to prioritize the treatment of cyber risks through a security management system. The impacts of cyber risks on countries’ economies and strategies became even clearer when the United States of America launched its National Maritime Cybersecurity Plan [6]. The Plan aims at reducing the potential catastrophic risks to national security and economic prosperity, considering the growing adoption of innovative digital technologies by organizations in the maritime sector.

Note that the integration of typical maritime systems with innovative digital technologies may open space for the development of novel attack strategies, which are not limited to those typically seen in the IT environment. The example of the alleged attack that occurred in the military Operation Orchard [7] can be brought to the discussion of cybersecurity of naval systems. In that operation, according to the literature [7,8,9], the Israeli air force attacked a Syrian facility without being noticed, thanks to a malicious cyber mechanism installed in the Syrian radar system. One hypothesis raised in the literature [7,8,9] regards to the possibility that an Electronic Attack (EA)—i.e., an attack performed in the electromagnetic spectrum—was used to unleash a cyberattack capable of hindering the radar computational process. The EA, in that case, would have been used to send commands to the Syrian air surveillance system, by using the radar receiver as an open door for attack commands. Not by chance, the concern about false data injection attacks in radar systems is gaining ground on the literature, such as in [10], where false information is injected into a networked radar system by an attacker with access to its communication links. Although the referred study is not focused on naval radars, it presents a data fusion algorithm to combat false data injection attacks in a generic networked radar system.

This said, considering the state of the art of ship systems, the example of the Operation Orchard [7] raises the question of whether naval sensors and information systems (e.g., radar systems and Automatic Identification Systems—AIS) can be used as open doors for an attacker to send attack commands to malwares hosted on the ship’s systems, at the attacker’s convenience. In the maritime sector, radar systems are used as relevant sensors for navigation safety or as a source of information for integrated navigation systems. Note that a compromised radar system may cause serious risks to the vessels’ safety, with possible impacts in a wide range of areas, as previously discussed. For this reason, it is important to study how such kind of attacks to radar systems can be implemented and seek for possible countermeasures.

The same concern applies to the AIS of ships. AIS transponders are designed to automatically provide position, identification and other information about a ship to other vessels and coastal authorities. Like radar systems, in today’s ship design, AIS are often interconnected to integrated navigation systems and other bridge technologies such as Electronic Chart Display and Information System (ECDIS). This integration allows AIS information from other vessels to be easily used to support decisions during navigation. On the other hand, the current AIS standard [11] lacks security mechanisms [3], which can make it prone to be exploited as an open door for cyberattacks.

Taking it into account, this paper presents a mechanism through which an attacker located outside the ship can use a radar system or an AIS as open door to remotely send commands to a cyber threat hosted in the vessel, even if the ship’s systems are not connected to other networks. The mechanism uses a template matching technique to detect specific patterns transmitted to the ship’s radar or AIS and, thus, use this information to trigger a malicious cyber process on the ship. The effectiveness of the proposed mechanism is assessed through simulations, where the target is either a generic radar system or an AIS/ECDIS setup. This said, the main contributions of this work are:

- The proposal of an attack concept where the radar antenna is exploited as open door for receiving malicious commands remotely sent to a cyber threat hosted on the radar computer. In this attack the malicious command is transmitted to the radar through an EA;
- The proposal of an attack concept where the AIS receiver is exploited as open door for receiving malicious commands remotely sent to a cyber threat hosted on an AIS/ECDIS setup (i.e., a navigation system where an AIS is connected to an ECDIS). In this attack the malicious command is transmitted to the AIS/ECDIS setup through forged AIS messages;
- The demonstration that a template matching technique is suitable to serve as a triggering mechanism capable of accurately acknowledging attack commands received and displayed in both radar PPI and ECDIS screens.

The rest of this paper is organized as follows: Section 2 presents the related works. Section 3 describes the mechanism proposed in this work to trigger cyberattacks in ships through AIS and radar systems. Section 4 presents the simulation results. Finally, Section 5 brings the conclusions.

## 2. Related Works

At the same time that maritime systems grow in automation and connectivity, the naval community is witnessing an upsurge in attacks and the exploitation of new attack vectors, making it necessary to expand research efforts to deny threats. In this context, this section presents related works focused on cybersecurity of radar systems, AIS and ECDIS of ships—which is the scope of this work.

In line with the new IMO standards [5], a structure is presented in [12] for assessing cyber risks that impact the navigation through analysis and detection of threats and vulnerabilities, penetration testing in networks, and specific analyzes for systems and critical assets. In [13], a comprehensive cyber risk assessment of a ship is carried out through an integrated experimental analysis encompassing vulnerability scanning in ECDIS with a software tool and interviews with crew. Based on the results, the authors generate a quantitative analysis of cyber risk on the ship.

In [14], the authors provide a summary on the main types of cyberattacks in the shipping industry, describe the main steps of the ship’s cyber risk assessment process, and briefly discuss general measures for mitigating cyberattacks on ships. Among the discussed cyber defense measures, the authors focus on means for ensuring physical cybersecurity, recommendations for mitigating risks in space-based and radio communication, email and browser protection, and the adoption of Intrusion Detecting System (IDS). Note that IDSs and other standalone attack detection methods [15] are extensively used in IT [16,17] and OT [18] networks and can bring benefits to the security of networked ship systems.

Referring to networked ship systems, ref [19] discusses security threats related to network communication in smart ships and proposes a secure ship network topology for the realization of autonomous ship operations. The proposed network topology enables secure communication by organizing the smart ship communication in three separated zones: Ship Global Zone (the top-level administrative network of the ship); Ship Control Zone (mission-critical systems, such as Integrated Bridge Systems and ECDIS); and Ship Systems Zone (which includes engine control, navigation sensors, and power systems).

The research described in [20] presents a study on the cybersecurity of integrated navigational system (INS), which interconnects ECDIS, AIS, radar systems, and other naval sensors. As in [13], the authors use a vulnerability scanner and interviews with the crew to examine the security resilience the ship. The results reveal cyber threats related to weaknesses of the INS operating system. The suggested countermeasures are twofold: preventive maintenance, and regulatory compliance. In [21], to study the origin of ECDIS cybersecurity risks, the authors examine of a set of ECDIS systems also using a vulnerability scanning software. The authors address threat detection in the ECDIS backup arrangement, operating system and third-party applications. Another analysis using a vulnerability scanner in an ECDIS is presented in [22]. The study concludes that even ECDIS systems approved with maintained ECDIS software can have vulnerabilities due to deficiencies in third party components.

In [23], the authors examine cybersecurity weaknesses in paperless ships that depend on two internetworked ECDIS workstations operated in a backup arrangement. As in [13,21,22] their method employs cyber security tests using a vulnerability scanning tool. The research found critical vulnerabilities caused by uncontrolled internetworking of unmaintained ECDIS workstations with identical hardware and software configurations.

In [3] the authors address the security requirements for autonomous and remotely controlled vessel, which are referred to as Cyber-Enabled Ship (C-ES). The research applies the Secure Tropos methodology to systematically elicit the security requirements of the three most vulnerable systems in a C-ES according to the authors: the Global Maritime Distress and Safety System (GMDSS), the Electronic Chart Display Information System (ECDIS) and the Automatic Identification System (AIS).

In [24], considering the growth of cyberattacks in the maritime sector, the authors describe a taxonomy that supports the creation of adversarial cyber models, risk mitigation and resilience plans applied to the maritime industry. The authors transfer the methods from the aviation sector to the maritime sector and demonstrate the approach using the AIS. Towards a secure AIS, ref [25] explores public identity-based cryptography and symmetric cryptography to improve the security properties of AIS. The approach brings benefits to integrated systems that use AIS data, such as e-navigation, e-bridging and autonomous ships. Another study to improve the cybersecurity of AIS is presented in [26]. The author presents the protected AIS (pAIS), which uses public key cryptography methods to provide mechanisms for authentication and message integrity and, thus, mitigate various AIS vulnerabilities.

Table 1 summarizes the main topics addressed by the related works and by the present study. Despite the aforementioned research efforts to characterize vulnerabilities and propose security solutions for ship navigation and information systems (e.g., ECDIS, AIS, integrated radar systems), to the best of the authors’ knowledge there is no work addressing how radar systems and AIS can be used as open doors to trigger cyberattacks hosted in a ship. In this sense, differently from the current literature, this work describes a triggering mechanism through which an attacker can activate a malicious code hosted in radar systems and ECDIS, even if these systems are not connected to computer networks located outside the ship.

## 3. Attack Triggering Mechanism

This section describes a method through which cyberattacks to naval sensors and systems can be remotely triggered using radar and AIS as open doors for receiving malicious commands. Section 3.1 presents the cybersecurity attack model. Section 3.2 describes the mechanism for triggering the cyberattack in a radar system, while Section 3.3 addresses how such an attack can be performed through an AIS system. Finally, Section 3.4 presents the details about the implementation of the triggering mechanism—used in both radar and AIS scenarios.

**Table 1:** Main topics addressed by the related works and by the present study.

	Systems					Main Contributions	
Ref.	Radar	AIS	ECDIS	INS	GMDSS	Studies on Attacks	Studies on Countermeasures
[24]		X				-	Describes a taxonomy to support the creation of adversarial cyber models, risk mitigation, and resiliency plans as applied to the maritime industry.
[12,13]			X			-	Proposes an analysis method that covers interviews with the ship’s crew and vulnerability scanning on ECDIS to identify threats and assess cyber risks.
[14]		X	X			Summarizes main types of cyberattacks in the shipping industry and their stages.	Discusses general measures for mitigating cyberattacks on ships: physical cybersecurity; recommendations for protecting radio systems; email and browser protection; and use of IDS in networked ship systems.
[19]	X	X	X			Discusses security threats related to network communication in smart ships.	Proposes a network topology that enables secure communication in smart ships, dividing the ship’s network into multiple zones.
[25]		X				-	Proposes a certificate-less Identity-Based Cryptography (IBC) along with pseudo-random Maritime Mobile Service Identity (MMSI) to enhance AIS security.
[3]		X	X		X	-	Applies the Secure Tropos methodology to systematically draw the security requirements of the three most vulnerable systems onboard a Cyber-Enabled Ship (C-ES), namely the AIS, the ECDIS and the GMDSS.
[20]				X		-	Presents a methodology that covers interviews with the ship’s crew and vulnerability scanning at the INS to identify threats and assess cyber risks on ships.
[21]			X			-	Identification of vulnerabilities in an ECDIS backup arrangement (in its underlying operating system and third-party applications) using the Nessus Professional scanning tool.
[22]			X			-	Presents an analysis of the cyber security weaknesses originating from the third-party components of the ECDIS software.
[23]			X			-	Assesses critical cyber threat vectors resulting from uncontrolled internetworking of unmaintained ECDIS workstations with identical hardware and software configurations.
[26]		X				-	Proposes the Protected AIS (pAIS): an implementation using public-key cryptography methods to address AIS security vulnerabilities.
Present study	X	X	X			Describes a method through which cyberattacks targeting naval sensors and systems can be remotely triggered using the ship’s radar or AIS as open door for receiving malicious commands.	-

### 3.1. Cybersecurity Attack Model

**Figure 1:**
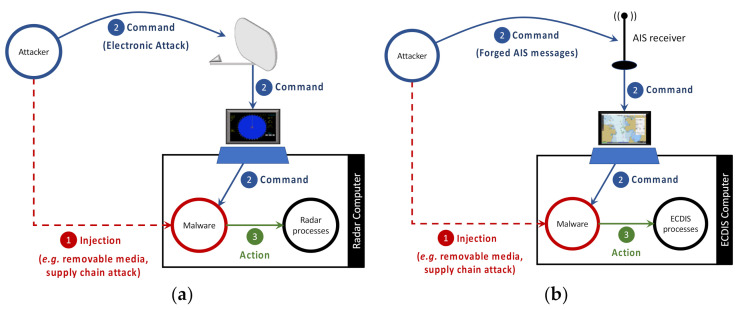
Attack model in: (**a**) a radar system; (**b**) an AIS/ECDIS setup.

The attack model discussed in this work considers a strategy where the attacker communicates with a cyber threat hosted in the ship’s systems through the radar antenna or the AIS receiver, as shown in Figure 1.

Note that it is possible to establish an analogy between the present attack model and the attack performed by bots that threaten network security. According to [27], a bot is a program that operates automatically as an agent for a user or program. In the context of cybersecurity, hackers forward bots to victims by several means infecting vulnerable computers. Once injected in the victim’s system, the bots then wait for commands from a hacker, who can manipulate them and the infected systems without the user’s knowledge [27].

The present attack model works in a similar way. It has the same lifecycle of a bot [28], which is divided in the three stages shown in Figure 1:

1. Malware Injection Stage: the attacker injects the malware in the target system (i.e., a radar computer or an ECDIS computer connected to an AIS receiver) by exploiting, for instance, removable media vulnerabilities [4,29] or even supply chain attacks [30,31];
2. Command Stage: the attacker remotely sends attack commands to the malware hosted on the target system. When attacking a radar system, the command is transmitted to the malware through an EA. When attacking an AIS/ECDIS setup, the command is transmitted using forged AIS messages. In this stage, the malware keeps monitoring the data received by the radar or AIS, seeking for a pattern corresponding to the attack command. If command is received and acknowledged by the malware, then the third stage (Action Stage) is triggered.
3. Action Stage: in this stage the malware manipulates the radar or ECDIS computational processes according to the command transmitted by the attacker. Examples of possible harmful actions performed in the targeted system during this stage are reset the system, record and replay scenarios, freeze the system display, etc.

The focus of this work relies in the second stage (Command Stage). Specifically, the focus is on the proposal and evaluation of the triggering mechanism used to acknowledge the received attack commands. The triggering mechanism must be accurate to ensure high probability in acknowledging commands and, at the same time, avoid accidental attack activations.

### 3.2. Triggering Mechanism in a Radar System

In [32], the authors discuss the concepts of hybrid attacks in the scope of sea power, where the cyber, electronic and kinetic warfare can be integrated to accomplish specific tactical and strategic purposes. The separate application of these kinds of warfare has been used in modern military operations, however it is noticed that there is a trend for these warfare dimensions to merge so that actions in one of them cause effects in the others. For example, in [33], the authors demonstrate a hybrid attack where an EA (more specifically a GPS spoofing attack) is able to produce a kinetic effect on a ship’s navigation.

Among the possible kinds of hybrid attacks discussed in [32], this section focuses on the cyber-electronic attack. Specifically, it addresses a particular attack against naval radar systems which, to the best of our knowledge, is not explored in the literature. According to [32], a cyber-electronic attack is an offensive where Electronic Warfare (EW) actions seek not only to manipulate the tactical information obtained through the electromagnetic spectrum (as in the traditional EW), but also to manipulate the computational process of the target system. In [34], the authors present an EA technique able to forge multiple false targets, with different ranges, within the radar detection range. The purpose of their technique is to produce multiple fabricated targets and, thus, make the radar operator unable to distinguish between the real target and the false targets. Note that in their case, the target detection information is manipulated, but the radar computational process continues to run normally. To make such EA able to manipulate the computational process, it would be necessary to have in the radar system a mechanism prepared to acknowledge the false information produced by the EA as a command to trigger the malicious cyber mechanism responsible for manipulating the system behavior.

Note that for such a cyber-electronic attack, it is necessary to have a cyber component previously implanted in the radar computing system, as shown in Figure 1. On this aspect, the literature report vulnerabilities implanted in air gapped systems (which is often the case of naval radar systems). These vulnerabilities can be implemented either in software, as in the Stuxnet [35,36,37,38], or in hardware through supply chain attacks, as in [8,39]. Special attention should be given to the operation Orchard. According to [8], commercial off-the-shelf microprocessors contained in the Syrian radar might have been purposely fabricated with a hidden hardware backdoor (referred to as kill switch) which, by receiving a preprogramed code had its functions disrupted and temporarily blocked the radar.

In this context, the aim of this section is to show—for awareness purpose—how the electronic and cyber warfare can be linked. As previously discussed, in [34] the authors present an EA able to produce multiple forged echoes for radar systems. In [8], the author presents clues about the implantation of a cyber vulnerability to affect radar systems, but not explain how such vulnerability can be triggered according to the convenience of the attacker, especially if radar computers are air gapped and the only path to send commands to a previously installed vulnerability is through the radar antenna. Here, we demonstrate a mechanism that can be used to link the electronic and cyber warfare domains—a key element for the construction of a cyber-electronic attack.

**Figure 2:**
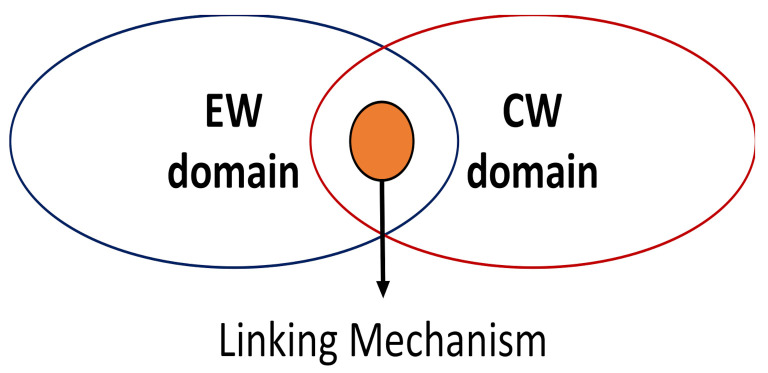
Linking mechanism between EW and CW domains.

In the attack addressed in this section, it is assumed that the electromagnetic spectrum is used by the attacker to send a sequence of forged pulses to the radar receiver, as defined in [34], which is coded in time/range to represent a command to the cyber mechanism hosted in the radar. As discussed in Section 3.1, once the command is acknowledged, the cyber component of the attack can start to manipulate the radar computational process to perform malicious actions, such as reset the system, stop to update the Plan Position Indicator (PPI), or even record and replay scenarios. The focus of this work is not on the generation of the forged radar echoes (an action executed in the EW domain in Figure 2), neither in the details about the manipulation of the radar computational process (an action executed in the Cyber Warfare domain, referred to as CW in Figure 2). The focus of this work is on the linking mechanism that lies between both domains to make a cyber-electronic attack feasible in a naval radar system.

**Figure 3:**
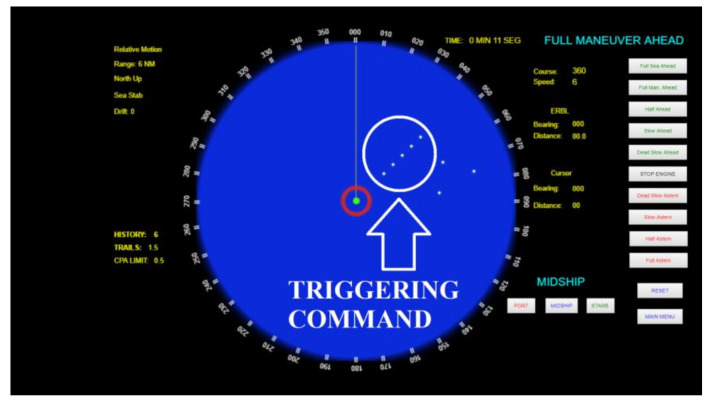
Representation of radar PPI screen with a triggering command (set of false echoes received due to an EA).

It is assumed that the cyber component of the attack (the malware) is already installed in the radar, given that the exploitation mechanisms to install it in the radar computational system is out of the scope of this paper. Additionally, considering that the implementation of the EA component of the attack is not in the scope of this paper, it is assumed that the remote command (a sequence of false echoes) is generated and transmitted through a Digital Radio Frequency Memory (DRFM) technique [34]. The command is received and processed by the radar, and displayed as an image in the PPI screen, such as any other received echoes (false or not). Figure 3 depicts an example that illustrates five false echoes, which can be produced by a DRFM-based EA (as proven in [34]), being displayed in the radar PPI screen.

**Figure 4:**
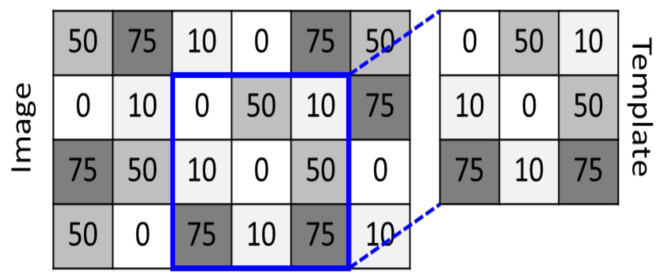
Example of a template matching.

Given the aforementioned scope, the linking mechanism herein proposed to make a cyber-electronic attack feasible in a naval radar system is based on a template matching technique [40]. The template matching technique is used in image processing to find small parts of an image that correspond to a model (template) image. To do so, it is defined a template to be searched in a main image. The main image in analysis and the template are divided in pixels, as shown in Figure 4. Then the template is moved over the main image, in a search process throughout all the main image’s area. For each position assumed by the template in this scanning process over the main image, a similarity index is computed. The similarity index quantifies the similitude between the template and the piece of the main image being compared. If the index is higher than a previously defined threshold σ, then the template image is considered to be detected in the main image. This search operation demands a computational cost proportional to the sizes of the images. On the other hand, it provides a high degree of effectiveness in searching for patterns in images [40].

Note that the degree of similarity between the template and a piece of the main image is established by comparing intensity values of each of their pixels. Among the available methods to compute the similarity coefficient, there are: the Sum of Absolute Differences (SAD), the Sum of Squared Differences (SSD), and normalized cross correlation. In this paper, the Pearson cross correlation (PCC) [40] is used (1):

(1) $$P=\frac{\sum_{i=1}^{N}\left(p_{i}-\bar{p}\right)\left(a_{i}-\bar{a}\right)}{\sqrt{\sum_{i=1}^{N}{\left(p_{i}-\bar{p}\right)}^{2}}\;\sqrt{\sum_{i=1}^{N}{\left(a_{i}-\bar{a}\right)}^{2}}}$$

wherein, $p_{i}$ is the intensity of pixel *i* in the template; p is the mean intensity of the pixels of the template; $a_{i}$ is the intensity of the pixel *i* in the patch of the image; $\bar{a}$ is the mean intensity of the pixels in the patch of the image; *N* is the number of pixels of the template (which must have the same dimensions as the patch of the image under analysis). Note that this method presents a normalizing term in the denominator, which gives it invariance to global changes in brightness [40], and the results always lie within a defined range [−1, 1].

Therefore, the linking mechanism herein proposed scans the radar PPI screen and uses the PCC (1) to identify a specific pattern corresponding to the triggering command sent to the radar through an EA. When the pattern is recognized in the PPI (i.e., when there is an image patch where P>σ), such match is used to unleash a pre-programed malicious action that manipulates the computational process of the radar (where the malware is hosted). Recall that, as previously discussed, the triggering command (i.e., the multiple false target displayed in the radar PPI) can be produced and transmitted by the attacker through a DRFM technique using the Sub-Nyquist sampling theorem [34], for instance.

### 3.3. Triggering Mechanism in a AIS/ECDIS System

The triggering mechanism described in Section 3.2 can also be applied to trigger a cyberattack in the context of an AIS connected to Electronic Chart Display and Information Systems (ECDIS). The AIS is a communication system used to provide data exchange in a maritime environment which contributes to the safety of navigation and facilitates traffic management. Ships and shore stations can share relevant information for a better situational awareness such as identity, position, time, course, speed, ship particulars, cargo, destination, navigation status, among other set of data. According to the International Convention for the Safety of Life at Sea (SOLAS), 1974 (as amended), all ships of 300 gross tonnage and upwards engaged on international voyages, cargo ships of 500 gross tonnage and upwards not engaged on international voyages and passenger ships irrespective of size are required to use AIS [41].

Originally, AIS data used to be visualized in a small LCD display located in its own equipment in a very unfriendly user interface, making it hard to operate. However, by using typical NMEA [42] gateway, ship’s navigation data bus can be used to transfer AIS data among all equipment inside a bridge environment and information can be input to navigational system devices such as radar display, ECDIS, Electronic Chart Systems (ECS) or Integrated Navigational Displays (INS) [41]. This approach made the use of AIS much more flexible and allowed a more comprehensive view of other ships on an electronic chart.

Navigational electronic equipment typically use an NMEA standard protocol to communicate and exchange a vast set of information called “Standard for Interfacing Marine Electronic Devices” [43]. NMEA sentences of the type “VHF Data-link Message” (VDM) are responsible for carrying AIS information inside the navigational data bus and are comma separated string sentences that transport in one of its blocks the original VHF transmitted AIS message (ITU-R M. 1371 radio message). This is a simple way to distribute AIS information to every navigational equipment on a bridge.

**Figure 5:**
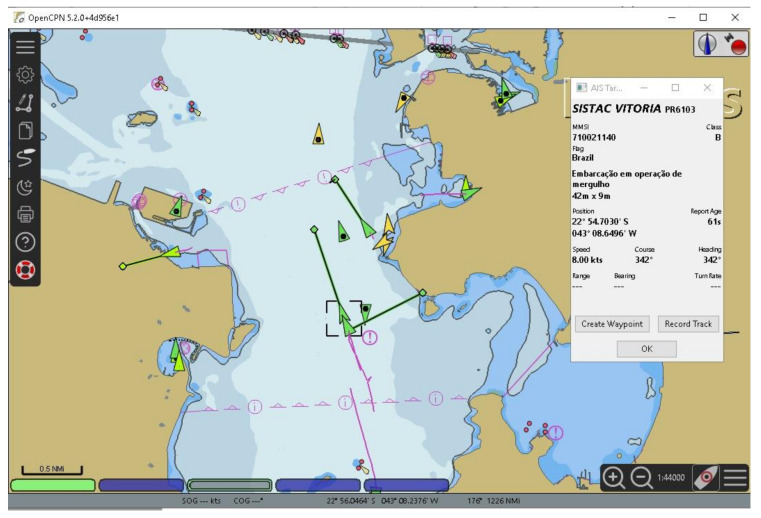
Example of a typical ECDIS screen displaying received AIS data.

To broadcast own ship information to other vessels, AIS stations communicate via VHF maritime mobile band using a time-division multiple access (TDMA) communications scheme described in ITU-R M.1371 [11]. In the AIS, there are 27 different types of messages used to exchange data between stations and the majority is concerned to navigational information. Messages Type 1 and Type 5 are the most relevant to officers in a bridge of a ship and represent important position and voyage related data report. By receiving Type 1 messages any ECDIS connected to ship’s navigation data bus will be able to plot the icon of the sender on top of an electronic nautical chart (as shown in Figure 5). Message Type 5 brings more relevant information about a ship in the vicinity called “static and voyage related vessel data report” sharing its type [43]. The shape of this plot on any ECDIS follows a rigid international standard described in IEC 62288 [44], so the AIS ships inside the VHF range (typically 20 Nautical Miles) can be shown in a harmonized way indicating its course, speed, navigational status, rate of turn, among many other relevant information for maritime situational awareness.

Despite the significant utility and importance of AIS to the safety at sea, these systems lack cybersecurity mechanisms and are prone to attacks. The literature demonstrates techniques where AIS data can be spoofed or hacked to malicious activities [24,25,45]. By forging and broadcasting false AIS messages, for instance [45], it is possible to insert false AIS plots in electronic navigation systems, such as an ECDIS. It means that the lack of security mechanisms in the AIS technology makes it prone to be used as an open door for receiving cyberattacks triggering commands, similarly to what is described in Section 3.1 in the scope of a radar system.

Here, to trigger a cyberattack through an AIS, the attacker first transmits a set of false AIS messages representing a set of ships in specific conditions (e.g., position, heading and speed). By receiving the forged messages, the ship’s AIS receiver will distribute the false messages in the ship’s navigation data bus among all equipment inside a bridge environment. Note that, an ECDIS connected to the navigation data bus will plot the false information on an electronic chart such as any other received AIS messages (false or not).

As in the attack described in Section 3.2, a malware hosted in the ECDIS scans the ECDIS screen and uses the PCC (1) to identify a specific pattern (the plot of a set of fake ships) corresponding to the triggering command sent by the attacker via AIS. When the pattern is recognized in the ECDIS screen (i.e., when there is an ECDIS image patch where P>σ), the attack code interprets the match as a command to unleash a pre-programed malicious action that affects the computational process of the ECDIS (where the malware is hosted).

**Figure 6:**
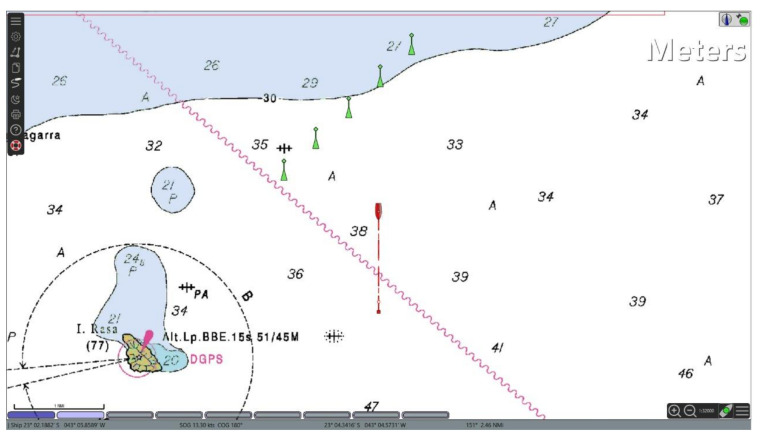
ECDIS screen displaying five false ships (green triangles with projected green lines) whose data was received through forged AIS messages.

Figure 6 exemplifies a scenario where an ECDIS displays five false ships plotted based on data received via false AIS messages. The false ships are represented by green triangles, where the respective velocity vectors are indicated by green lines projected from the ship position. Here, the triggering command consists of the graphical pattern produced by the plot of these five false ships whose data were received via AIS. Note that, in this case, the template used as reference by the triggering mechanism hosted in the ECDIS is a graphic representation of these fake ships with specific positions and velocity vectors. It is worth recalling that the symbols used to represent the false ships (as any other ship, fake or not) in any ECDIS follows a rigid international standard (i.e., IEC 62288 [44]). It allows the template matching technique to be effective regardless of what specific ECDIS the attack code is deployed.

### 3.4. Implementation of the Triggering Mechanism

**Figure 7:**
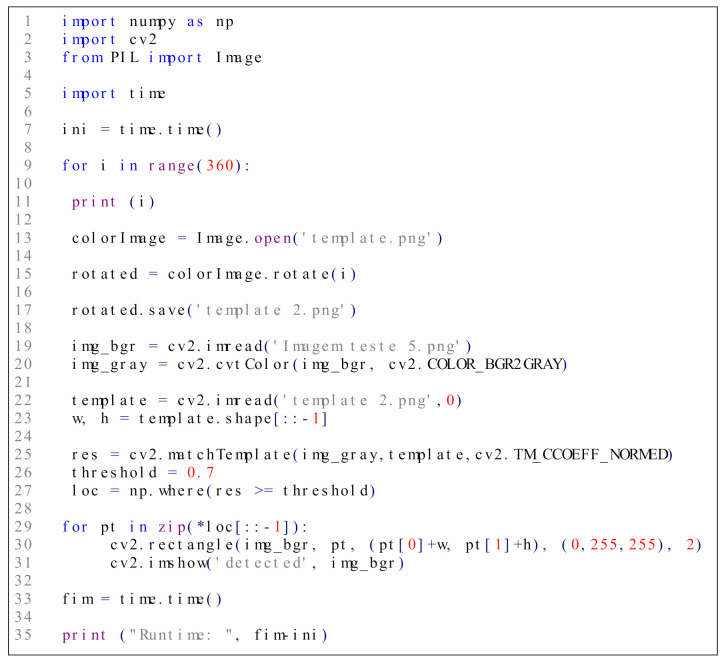
Triggering mechanism implementation in Python.

**Figure 8:**
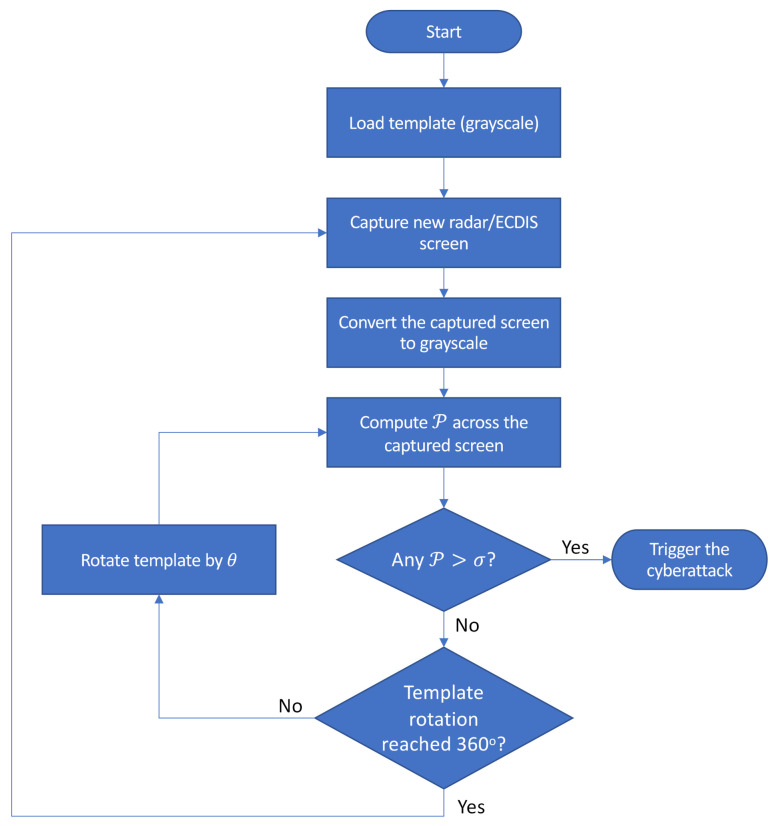
Flowchart of the triggering mechanism.

The triggering mechanism herein proposed as a method to receive commands and unleash cyberattacks in radar systems and AIS/ECDIS was implemented on a computer endowed with an Intel i7 processor of 2.5 GHz, 8G RAM DDR3 memory, running Microsoft Windows 10, 64 bits. The template matching mechanism (i.e., the image scan routine and the PCC computation to verify whether a P>σ is found in the image) was implemented in Python using the libraries NumPy, cv2 and Pillow. The implementation code is shown in Figure 7. For a better understanding of the entire process executed by the triggering mechanism, Figure 8 shows its flowchart.

As shown in Figure 8, during the search process, the test image (i.e., the radar PPI screen or the AIS/ECDIS screen) is read and converted to grayscale. This serves to eliminate possible color variations, performing only the analysis of the pixel intensity. The template is also processed in grayscale for the comparison. Additionally, it is necessary to consider that the triggering command can be displayed in different orientations, depending on the display mode of the radar PPI or ECDIS screen (e.g., north-up, head-up, course-up). In the case of a radar system, the triggering command can also be displayed in different orientations depending on the Angle of Arrival (AOA) of the EA. Therefore, during the search process for a match, the template is iteratively rotated by θ degrees, until its rotation reaches 360, as described in Figure 8.

Note that the same triggering mechanism implementation is used in both radar and AIS/ECDIS scenarios, differing only in the templates used due to the differences between a radar PPI plot and an AIS/ECDIS plot. The radar environment is simulated in the Cinematic Radar Simulator v.2.0. The AIS/ECDIS scenarios are generated using the open source navigation software OpenCPN version 5.0.

## 4. Results

This section presents the simulation results of the attacks described in Section 3. Section 4.1 reports the results on the performance of the triggering mechanism in a radar system. Section 4.2 reports its performance in an AIS/ECDIS system.

### 4.1. Simulations of the Radar-Based Attack

In the simulations herein presented, the Python code presented in Section 3.3 scans the graphical interface (the radar PPI) produced by the radar simulator in order to identify the attack commands received from the EA component of the offensive. To evaluate the effectiveness of the proposed mechanism, the chosen attack command consists of a sequence of five false echoes (such as in [34]), which produces a sequence of five points displayed in the direction where the DRFM transmitter is (such as represented in Figure 6). Once this pattern is detected, it can be used to trigger a malicious action in the naval radar system.

**Figure 9:**
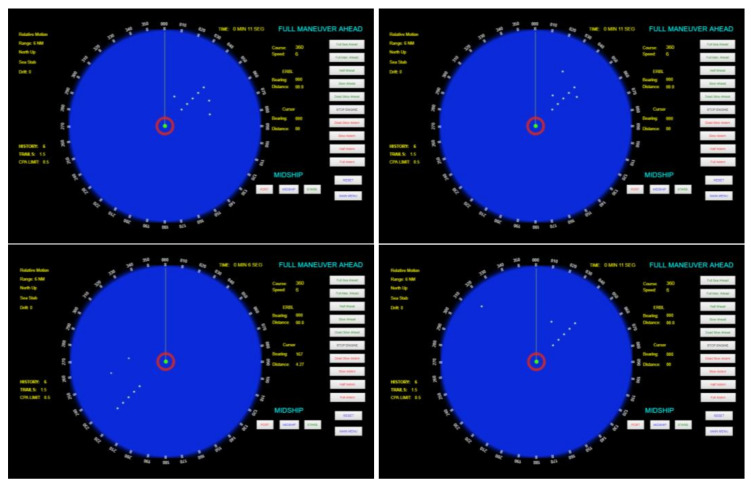
Examples of real positive cases where the attack command is displayed in the radar PPI.

**Figure 10:**
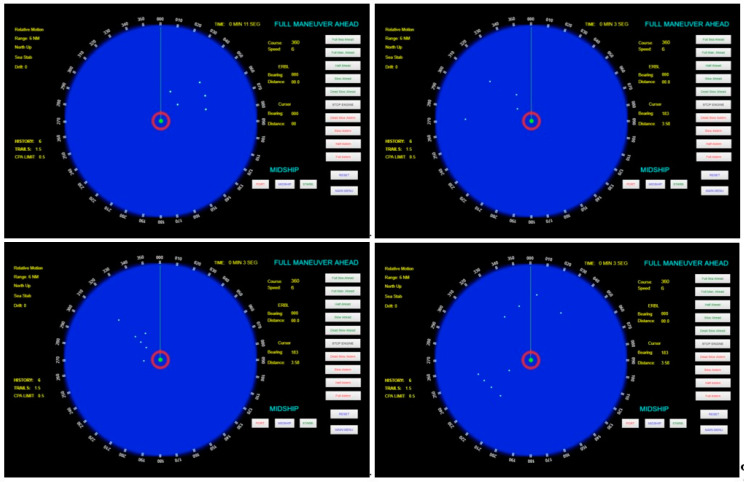
Examples of real negative cases where the attack command is not displayed in the PPI.

To validate the mechanism and test the effectiveness of the command detection method, 30 fictitious scenarios were generated using the radar simulation software in order to represent real situations where a naval platform could be. Clutter/target echoes that might affect the command detection were randomly inserted in the PPI. Figure 9 shows examples of real positive cases used in the simulations, where it is possible to observe the presence of the attack command (the sequence of five aligned false echoes). Figure 10 shows examples of real negative cases used, where the attack command is not present in the radar PPI.

It is worth mentioning that the attacker is transmitting the EA signal that generates triggering command shown in the screen, and that the signal can come from any direction, depending on the DRFM transmitter location. Thus, it is necessary to consider different angles from which the triggering command could be received. For the sake of simplicity, variations of 1 degree are considered, so the attacker could emit from the directions 000, 001, 002, 003 and so on. Considering these possible different Angles of Arrival (AOA), the template containing the triggering command pattern is also rotated in steps of 1 degree during the search process throughout the PPI. This template matching search is executed throughout all the PPI screen until the algorithm finds a match (i.e., a P>σ) or until all possibilities along the screen are tested.

**Figure 11:**
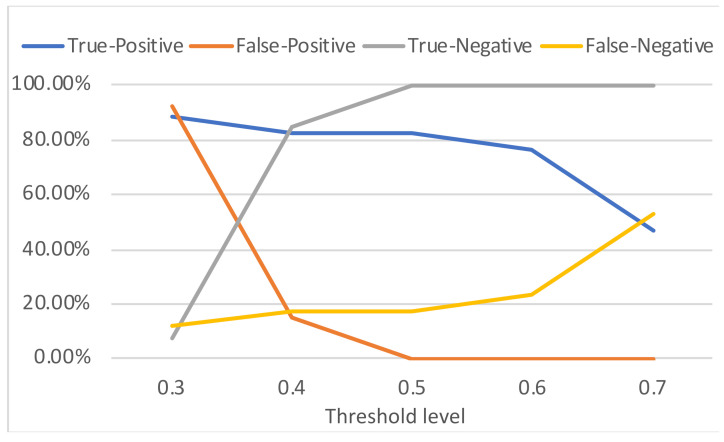
Performance of the triggering mechanism in attacks to a radar system.

**Figure 12:**
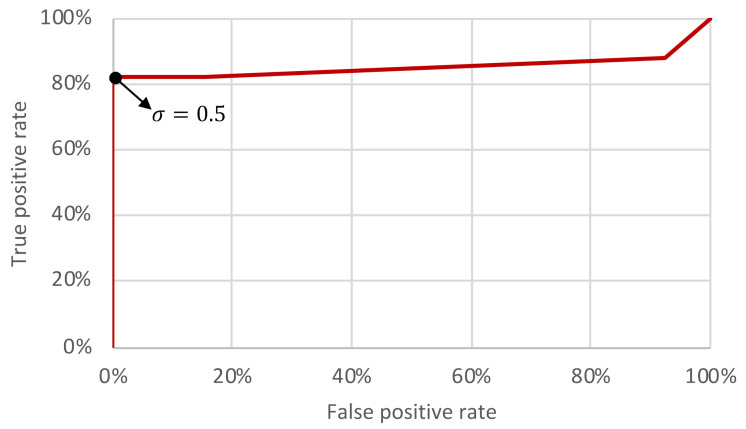
ROC curve of the triggering mechanism in a radar system.

Five threshold levels σ were assessed: 0.3, 0.4, 0.5, 0.6, 0.7. Recall that the computed PCCs are compared with the threshold levels in order to decide if a match was found or not (see Section 3). Each threshold level was assessed using the set of 30 different scenarios. The confusion matrices and accuracies of each threshold level are compiled in Table 2. The performance rates (in percentages) of the triggering mechanism for each threshold level is depicted in Figure 11 and the obtained receiver operating characteristic (ROC) curve is shown in Figure 12.

The situation of a True-Positive (TP) refers to the case where the triggering command is present in the PPI and there is a match with the template. A False-Positive (FP) is the case in which the triggering command is not present in the PPI, but there is a match with the template. A True-Negative (TN) occurs when the triggering command is not present in the PPI and is there is no match with the template. Finally, the False-Negative (FN) occurs when the triggering command is in the PPI, but it is not detected.

The area under the ROC curve shown in Figure 12 is 0.8527, which indicates that the method is adequate to act as a mechanism to recognize the received commands and trigger a cyberattack hosted in a radar system. Based on the results, lowering the threshold increases the TP rate, but also increases the FP rate (which may cause fortuitous and unwanted attack activations). On the other hand, increasing the threshold decreases the FP rate, but also decreases the TP rate (which reduces the attack effectiveness). According to Figure 11 and Figure 12, the best threshold from the attacker point of view is 0.5—where the accuracy is 0.90 as shown in Table 2. Note that with this threshold the attacker is able to obtain the maximum TP rate (82.35%) without false positives. It means that, with this threshold, considering the evaluated scenarios, the probability of an accidental attack activation tends to 0% (which is important to avoid the attack disclosure) and the attacker has 82.35% of probability in successfully activating the cyber component of the attack in the first attempt. Note that, with two attempts the probability of having the attack properly activated in at least one of the attempts increases to 96.88%.

### 4.2. Simulations of the AIS-Based Attack

**Figure 13:**
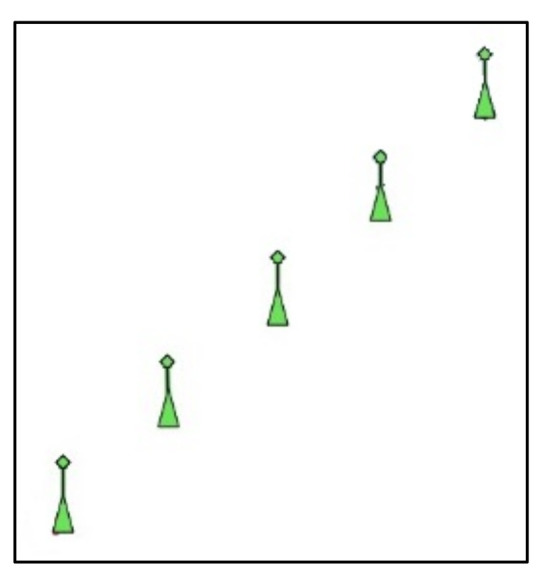
Template with five false AIS plots.

This section evaluates the performance of the proposed triggering mechanism in scenarios where the triggering command is received via AIS and displayed in an ECDIS, as described in Section 3.3. The Python code shown in Section 3.4 scans the ECDIS screen in order to search and identify the attack command—i.e., the false AIS data plotted in the electronic chart. In this simulation, the chosen attack command consists of a sequence of five false AIS plots aligned with each other and with the same velocity vectors, as shown in Figure 13. As previously discussed, once such pattern is detected, it can be used to trigger a malicious action in the ECDIS.

To validate the mechanism and test its performance, 30 different scenarios were generated using the OpenCPN v. 5.0 in order to represent real situations where the ship could be. The nautical chart used in the simulations is the raster chart 1501, available in [46]. Note that the electronic chart in the background (as shown in Figure 6) represents a noise to the template matching process, given that the template contains only the false AIS plots and a white background. In each scenario, the own ship and the set of false AIS plots were placed in different positions on the electronic chart.

**Figure 14:**
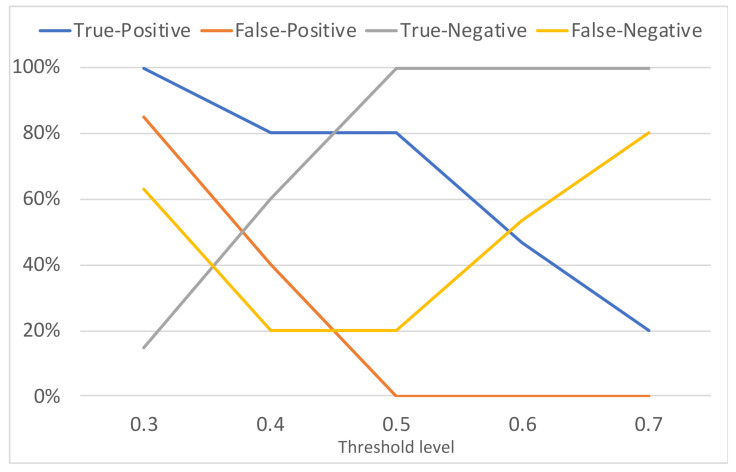
Performance of the triggering mechanism in attacks to an AIS/ECDIS system.

**Figure 15:**
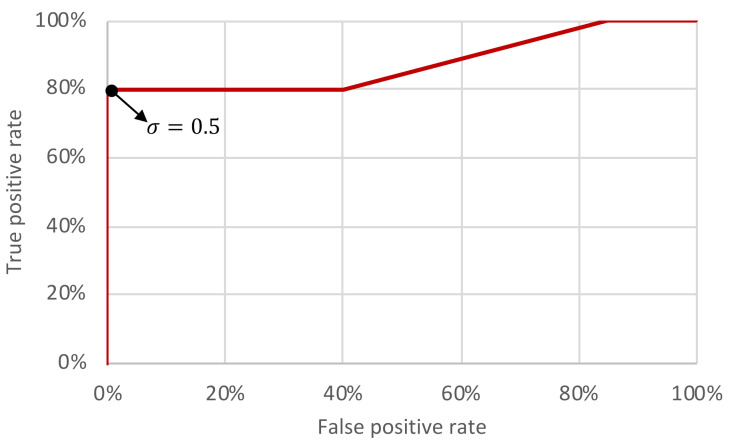
ROC curve of the triggering mechanism in an AIS/ECDIS setup.

This template matching search is executed throughout all the ECDIS screen until the algorithm finds a match (i.e., a P>σ) or until all possibilities along the screen are tested. As in the simulations of the radar-based attack, five threshold levels σ are assessed: 0.3, 0.4, 0.5, 0.6, 0.7. Recall that, as described in Section 3, the PCCs are compared with the threshold levels σ in order to decide if a match is found or not. Each σ is assessed using the set of 30 different scenarios. Table 3 compiles the confusion matrices and accuracies obtained using each threshold level. The performance of the triggering mechanism for each threshold level is also shown in Figure 14 and the obtained ROC curve is shown in Figure 15.

The results of the simulations in the AIS/ECDIS system present a profile similar to that obtained in the simulations in a radar system (see Section 4.1). The area under the ROC curve provided in Figure 15 is 0.8750, which indicates that the method is also adequate to act as a mechanism to trigger cyberattacks in an AIS/ECDIS setup. It is possible to see in Figure 14 and Table 3 that the reduction of the threshold σ makes the TP rate to increase, but also increases the FP. When σ=0.3, for instance, the FP rate is 85%. Note that high FP rates are not desired, as an FP can cause accidental and unwanted attack activations. On the other hand, the increase of σ causes the FP rate to decrease, but also reduces the TP rate (which lowers the effectiveness of the attack). As in the simulations on the radar PPI, the results show that the best threshold level in the attacks to the AIS/ECDIS is σ=0.5 where, as indicated in Table 3, the accuracy is 0.93. In this case, according to Figure 14 and Figure 15, the attacker achieves the maximum TP rate (80%) without FP. In other words, for σ=0.5 the probability of having an accidental attack activation in the AIS/ECDIS tends to 0% (which prevents the untimely disclosure of the attack) and the attacker has 80% of probability to successfully activate the cyber component of the attack in one attempt. With two attempts, the probability of having the attack properly activated (i.e., have the attack command recognized by the triggering mechanism) in at least one of the attempts increases to 96%.

Note that when σ=0.5 the TP rate in the AIS/ECDIS (80%) is approximately the same as in the radar (82.35%). The slightly lower performance in the AIS/ECDIS is due to the background noise caused by the electronic chart. The results show that the effect of the noise caused by the electronic chart in the TP rate is more accentuated in the AIS/ECDIS simulations when σ>0.5. In these cases, the TP rate decreases faster in the AIS/ECDIS simulations than in the radar simulations.

**Figure 16:**
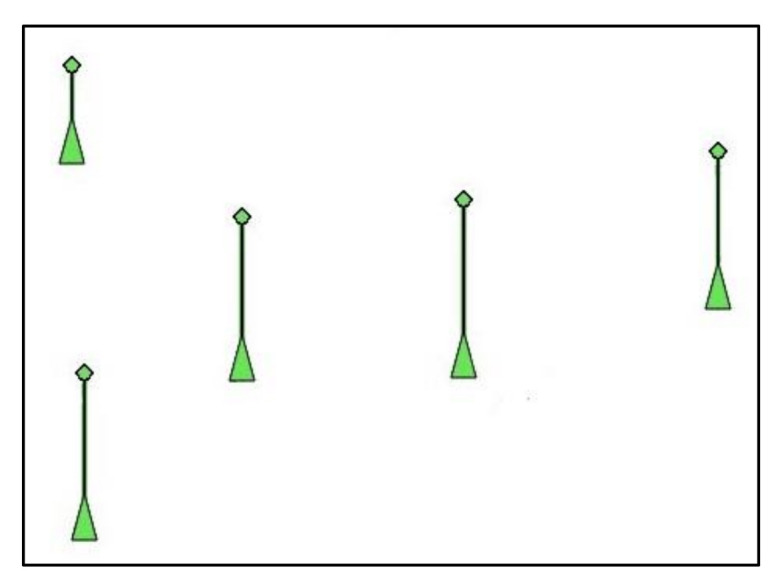
Template with five false AIS plots randomly distributed.

Despite the effectiveness and accuracy observed in the results provided by the attack command shown in Figure 13 (five false AIS plots aligned with each other), it worth mentioning that this is a pattern not commonly produced by ships at sea. It is effective in triggering the cyberattack, but could draw the attention of a watchful mariner. In this sense, the method is also evaluated using a different and more irregular pattern of AIS plots as an attack command. To exemplify and demonstrate the effectiveness of the triggering mechanism in this condition, the attack command shown in Figure 16 is used, where five false AIS plots are randomly distributed in the template area using a uniform distribution.

**Figure 17:**
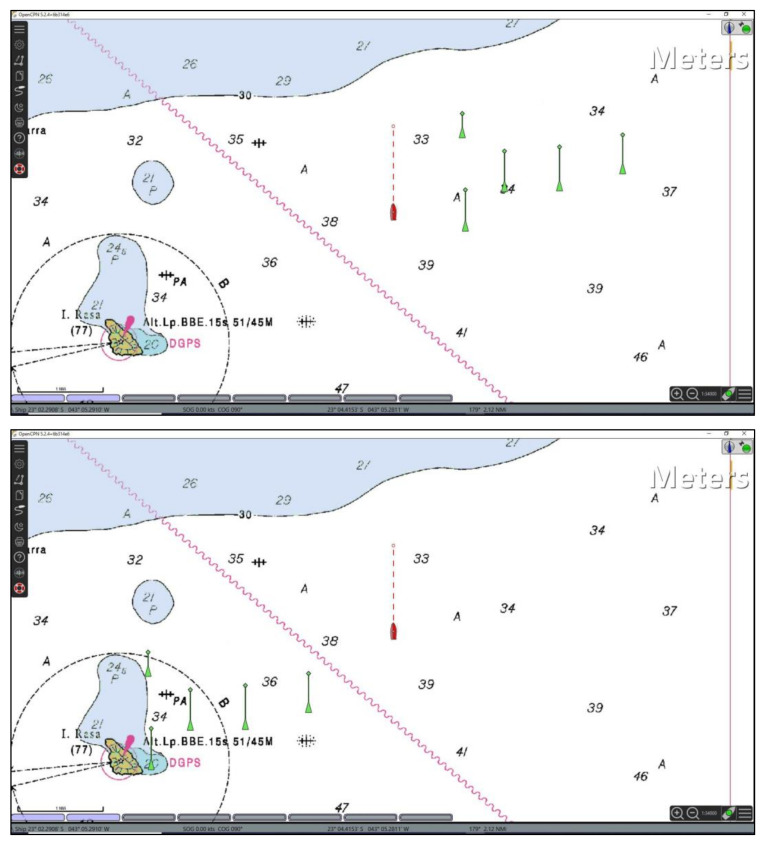
Examples of scenarios where the attack command is the set of five false AIS plots shown in Figure 16 (randomly distributed).

The performance in detecting the attack command shown in Figure 16 is evaluated using 30 scenarios considering the threshold level that achieved the best accuracy in Table 3 (σ=0.5). The scenarios containing the attack command are produced by randomly placing false AIS plots (i.e., the template shown in Figure 16) in different areas of an electronic chart. The location where the template is placed on the electronic chart is defined using a uniform distribution. Figure 17 exemplifies scenarios containing the attack command shown in Figure 16.

Table 4 presents the confusion matrix and accuracy obtained in the simulations using the template of Figure 16 as an attack command. Note that the achieved accuracy (0.93) is the same obtained in the simulations shown in Table 3 for σ=0.5. It means that the effectiveness of the triggering mechanism using a randomly generated template is the same as the effectiveness obtained when the template is a set of false AIS plots aligned, but less likely to attract the attention of the mariner. Note that sending the attack command using forged AIS messages provides more flexibility do introduce random plots in the ECDIS screen than in the case of an attack on a radar system. In the former, the position of the false AIS plots can be easily configured in the AIS messages transmitted by the attacker. On the other hand, when the target is a radar system, its antenna rotation increases the difficulties to insert randomly distributed plots in the PPI (i.e., false plots not only with different ranges, but also with different bearings).

## 5. Conclusions

This work presents a triggering mechanism through which an attacker located outside the ship can use a radar or an AIS as open door to remotely send commands to a cyber threat hosted in the vessel, even if the ship’s systems are air gapped—i.e., not connected to other networks. The mechanism uses a template matching technique to recognize specific patterns plotted in the radar PPI or ECDIS screen. By recognizing these patterns (attack commands remotely transmitted by the attacker) a malicious code hosted in ship’s radar or ECDIS can initiate harmful preprogramed actions (e.g., reset the system, or freeze the radar or ECDIS screens).

Considering the theoretical framework and the simulation results presented in this paper, it is possible to realize that the proposed mechanism enables the use of radar systems and AIS as open doors for activating cyberattacks in ships’ systems. The triggering mechanism is able to recognize the attack commands with good effectiveness, maintaining the due safety against accidental activations. In the best case, the TP rate in the AIS/ECDIS is 80% and in the radar system is 82.35%, considering one attack attempt. With two attempts, the probability of having the attack properly activated in at least one of the attempts increases to 96% in the AIS/ECDIS, and to 96.88% in the radar system. Note that, at the same time that these TP rates are achieved, the FP rate provided by the triggering mechanism is 0% (which is important to avoid accidental attack activations).

For future work we plan to investigate countermeasures to mitigate this threat—such as tools to verify the integrity of the software used in naval radar systems and ECDIS.

## Figures and Tables

**Table 2:** Confusion matrix and accuracy of each threshold level in the attack to a radar system.

		Actual Condition		
		Positive	Negative	Accuracy
σ=0.3	Predicted positive	15	12	0.53
	Predicted negative	2	1	
σ=0.4	Predicted positive	14	2	0.83
	Predicted negative	3	11	
σ=0.5	Predicted positive	14	0	0.90
	Predicted negative	3	13	
σ=0.6	Predicted positive	13	0	0.86
	Predicted negative	4	13	
σ=0.7	Predicted positive	8	0	0.70
	Predicted negative	9	13	

**Table 3:** Confusion matrix and accuracy of each threshold level in the attack to an AIS/ECDIS setup.

		Actual Condition		
		Positive	Negative	Accuracy
σ=0.3	Predicted positive	10	17	0.43
	Predicted negative	0	3	
σ=0.4	Predicted positive	8	8	0.66
	Predicted negative	2	12	
σ=0.5	Predicted positive	8	0	0.93
	Predicted negative	2	20	
σ=0.6	Predicted positive	7	0	0.90
	Predicted negative	3	20	
σ=0.7	Predicted positive	3	0	0.76
	Predicted negative	7	20	

**Table 4:** Confusion matrix and accuracy of the attack to an AIS/ECDIS setup using the template shown in Figure 16.

		Actual Condition		
		Positive	Negative	Accuracy
σ=0.5	Predicted positive	13	2	0.93
	Predicted negative	0	15	

## Data Availability

The data presented in this study are available on request from the corresponding author.
